# Absorption of Cataract—One Year after First Discission, and Ten Months after Second

**Published:** 1886-11

**Authors:** T. J. Tyner

**Affiliations:** San Antonio, Texas


DANIEL'S

### Texas Medical Journal.

PUBLISHED MONTHLY.—SUBSCRIPTION $2.00 A YEAR.

Vol. 2. AUSTIN, NOVEMBER, 1886. No. 5.

### ORIGINAL ARTICLES.

☞CONTRIBUTED EXCLUSIVELY TO THIS JOURNAL.☜

*The Articles in this Department are accepted and published with the understanding that we are not responsible for, nor do we indorse the views and opinions of the writers by so doing.*

### ABSORPTION OF CATARACT.

(PROBABLY CONGENITAL) ONE YEAR AFTER FIRST DISCISSION AND TEN MONTHS AFTER SECOND.

*Reported by T. J. Tyner, M. D., San Antonio, Texas.*

(For Daniel's Texas Medical Journal.)

MISS R., age 19. Health and family history good. Has never had either convulsions or any serious illness. As well as she can remember her vision has never been good, although has been able to read until six years ago. In right, vision = 15–200 in left 2–200.

July 1st, 1885, with the application of a four per cent solution of cocaine, and without pain, I made a discission in left eye, rotating the needle in the lens substance. There was much swelling but no reaction. The pupil was kept well dilated with atropine and instead of the bandage I used cold compresses with solution of boric acid.

September 1st following, there being no perceptible indication of absorption, I made a second operation, dividing the lens in several places; flakes of which in two or three days dropped down in the lower part of anterior chamber. Notwithstanding she was taken during the night with an attack of dengue fever which was quite severe, no reaction whatever followed, and the lens substance in the anterior chamber disappeared in two or three weeks, by absorption.

June 1886. I had seen the young lady from time to time, and had given the operation up as a failure, and so stated in Dr. Geo. Cupples' report on Texas Surgery, and contemplated extracting the cataract, as the other eye had grown practically useless; but to my surprise and gratification she presented herself to-day, with the lens absorbed, except a small area in lower segment. A perfect view of the fundus was obtained, and vision = 15–70.

November fifth, vision has increased to 15–30 partly, with a probability of still further improvement. There is not the slightest stain or opacity of either the anterior or posterior capsule. No synechia, and the pupil reacts thoroughly.

The object of interest in this case, (and the only one), is the great length of time required for absorption—nearly one year. Absorption is usually complete in children in from four to ten weeks, and in young adults from six or seven weeks to three months, rarely ever going beyond that time.

I shall operate in the other eye in a few days, and will give the result to the readers of the JOURNAL.

There was an outside feature in the case worthy of mention. Atropia was applied sufficiently often to keep the pupil dilated, but a few weeks after the second operation it produced lachrymation, hyperæmia, increased tension and intense pain, lasting eight to twelve hours. As these symptoms increased in intensity after each application, the use of the drug was discontinued.

San Antonio, November 7. 1886.

